# Interplay between Epigenetics and Cellular Metabolism in Colorectal Cancer

**DOI:** 10.3390/biom11101406

**Published:** 2021-09-25

**Authors:** Xiaolin Zhang, Zhen Dong, Hongjuan Cui

**Affiliations:** 1 Cancer Center, Medical Research Institute, Southwest University, Chongqing 400716, China; zxl19991124@email.swu.edu.cn; 2 State Key Laboratory of Silkworm Genome Biology, Institute of Sericulture and Systems Biology, College of Sericulture & Textile & Biomass Science, Southwest University, Chongqing 400716, China; 3 Engineering Research Center for Cancer Biomedical and Translational Medicine, Southwest University, Chongqing 400716, China; 4 Chongqing Engineering and Technology Research Center for Silk Biomaterials and Regenerative Medicine, Southwest University, Chongqing 400716, China

**Keywords:** colorectal cancer, epigenetics, cellular metabolism, targeted therapy, tumorigenesis

## Abstract

Cellular metabolism alterations have been recognized as one of the most predominant hallmarks of colorectal cancers (CRCs). It is precisely regulated by many oncogenic signaling pathways in all kinds of regulatory levels, including transcriptional, post-transcriptional, translational and post-translational levels. Among these regulatory factors, epigenetics play an essential role in the modulation of cellular metabolism. On the one hand, epigenetics can regulate cellular metabolism via directly controlling the transcription of genes encoding metabolic enzymes of transporters. On the other hand, epigenetics can regulate major transcriptional factors and signaling pathways that control the transcription of genes encoding metabolic enzymes or transporters, or affecting the translation, activation, stabilization, or translocation of metabolic enzymes or transporters. Interestingly, epigenetics can also be controlled by cellular metabolism. Metabolites not only directly influence epigenetic processes, but also affect the activity of epigenetic enzymes. Actually, both cellular metabolism pathways and epigenetic processes are controlled by enzymes. They are highly intertwined and are essential for oncogenesis and tumor development of CRCs. Therefore, they are potential therapeutic targets for the treatment of CRCs. In recent years, both epigenetic and metabolism inhibitors are studied for clinical use to treat CRCs. In this review, we depict the interplay between epigenetics and cellular metabolism in CRCs and summarize the underlying molecular mechanisms and their potential applications for clinical therapy.

## 1. Introduction

Colorectal cancer (CRC), a multi-step, multi-factor complex disease, is a common malignant tumor of the digestive tract. It has become the world’s third most common cancer and the second most common cause of death from cancer [1]. Both genetic and environmental risk factors play essential roles during the development of CRC [2]. In recent years, there have been large advances in cancer diagnosis technology. However, because the early symptoms are not dominant, most patients are diagnosed at an advanced stage, which affects the prognosis. Therefore, it is urgent to reduce the high incidence and mortality of CRC, and to develop advanced prevention and treatment strategies. For decades, people have elucidated many aspects of CRC biology, including genetics, epigenetics, metabolism and signaling pathways. Among them, epigenetic changes and cellular metabolic reprogramming play a key role in the occurrence and development of CRC, which is becoming more and more important.

Epigenetics refers to the regulatory code that dictates gene expression or not and can be stably inherited in the absence of a constant genomic sequence. The current research content of epigenetics mainly includes DNA methylation and hydroxylmethylation, histone modifications, chromosome remodeling, and non-coding RNA regulation. In the early stage of CRC, DNA methylation status begins to change abnormally, mainly through the hypermethylation of some CpG islands leading to the down-regulation of gene expression and genome-wide hypomethylation, which cause genome instability to participate in tumorigenesis and development [3,4]. Histone modifications are often closely related to DNA methylation and act together in the process of gene transcription. Covalent modifications in histones includes acetylation, methylation, phosphorylation, ADP-ribosylation, ubiquitination, succinylation and myristoylation, etc. Histone methylation modifications mainly include the monomethylation, dimethylation or trimethylation of histone H3 or H4, such as H3K4me2, H3K4me3, H3K9me3 and H3K27me3. Histone acetylation modifications mainly include histone H3 and H4 acetylation. Histone modification significantly affects gene expression; therefore, there are obvious histone modification abnormalities in CRC [5]. MicroRNA (miRNA) is a small single-stranded non-coding RNA that can regulate the expression of various oncogenes and tumor suppressor genes after transcription. The dysregulated expression of many miRNAs has been shown to mediate important signaling pathways in the multi-step carcinogenesis of CRC [6]. In recent years, long non-coding RNA (lncRNA) has also been shown to be highly associated with the tumorigenesis of CRC [7,8]. Even in mitochondrion, epigenetics also plays a fundamental role in energy hemostasis [9].

Cell division and proliferation requires a large amount of protein, lipid and nucleic acid as molecular raw materials and adenosine triphosphate (ATP) as energy, leading to the reorganization of anabolic flow in tumor cells. Metabolic reprogramming contributes to tumor progression and metastasis, which is considered to be an important hallmark of cancer [10]. The most dominant metabolic reprogramming is the aerobic glycolysis, also called the Warburg effect. Tumor cells increase glucose consumption to promote glycolysis, converting pyruvate to lactic acid even under aerobic conditions, which is different to that of the normal cells. However, glutamine metabolism and oxidative phosphorylation (OXPHOS), as well as lipometabolism and one-carbon pathways, are also altered in some tumor cells because of genetic heterogeneity and microenvironmental discrepancy [11,12,13,14,15]. Cellular metabolism is exquisitely modulated. Nonetheless, the regulatory axis in tumors are usually disorganized. This is the reason why dysregulated cell metabolism is observed in tumors. Cell metabolism is one of the essential factors contributing to carcinogenesis, and is also a result of malignancy [16,17]. Studies have found that metabolic reprogramming is widespread in CRC. In different types of cancers, including CRC, dysregulated metabolic pathways such as abnormal glycolysis, glutamate and lipid synthesis are often observed, leading to unlimited tumorigenesis [18].

It is worth noting that certain metabolic changes are known to occur at the epigenetic level, so that epigenetics and metabolism are highly intertwined in a mutually beneficial manner [19]. Metabolites produced in metabolic pathways, such as glycolysis cycles and OXPHOS, can be used as cofactors for epigenetic regulation [20]. Studies have also shown that epigenetics can regulate the expression of metabolism-related genes and affect the metabolic reprogramming of tumor cells [21,22,23]. Since many metabolic changes and epigenetic regulation are common in multiple cancer types, they become promising targets for tumor therapy. The purpose of this review is to describe the interactions between epigenetic changes and metabolic reprogramming, and to list the research progress of drugs targeting epigenetics and metabolism in CRC.

## 2. Epigenetics in CRC

### 2.1. DNA Methylation and Hydroxymethylation in CRC

#### 2.1.1. DNA Methylation in CRC

DNA methylation is the covalent bonding of a methyl group at the 5′ carbon position of the cytosine of CpG dinucleotides under the action of DNA methyltransferases (DNMTs) to form 5-methylcytosine (5mC). DNMTs are mainly divided into two categories: DNA methylation maintenance enzyme DNMT1 and DNA de novo methylase DNMT3A, DNMT3B and DNMT3L [24]. In the human genome, CpG dinucleotides account for 10% and exist in two forms. One is dispersed in DNA and accounts for 70% to 80% of CpG dinucleotides, and this form is in the form of methyl. Another form, where CpG sites are highly clustered, is called CpG islands, which only account for 1% to 2% of the genome. They are mostly found in the 5′ promoter region of the gene, and can also extend to the exon region of the gene. Changes in normal DNA methylation patterns include DNA hypomethylation, which occurs in normal unmethylated regions of the genome, and DNA hypermethylation, which usually occurs on CpG islands [25].

Recent studies have shown that inactivation of multiple tumor-suppressor genes due to abnormal methylation of CpG islands plays an important role in the development of CRC [26]. DNA methylation has a unique subtype in CRC, which is called the CpG island methylation phenotype (CIMP) [27]. CIMP has been considered a significant direction for CRC research [28]. Evidence shows that this abnormal methylation has been found in the promoter regions of some vital tumor suppressor genes in CRC, including O6-methylguanine-DNA methyltransferase (MGMT), thrombospondin 1 (THBS1), tissue inhibitor of metalloproteinases 3 (TIMP3), p14^ARF^, p15^INK4B^ (cyclin dependent kinase inhibitor 2B), p16^INK4a^, adenomatous polyposis coli (APC), deleted in colorectal carcinoma (DCC), MutL homolog 1 (MLH1), insulin-like growth factor 2 (IGF2), suppressor of cytokine signaling 1 (SOCS-1) and Runt-related transcription factor 3 (RUNX3) genes. The CpG island methylation in the promoter region of the above genes may play a crucial role in the occurrence and development of CRC, and the hypermethylation of the p16 promoter region is common in CRC. Moreover, hypermethylation of the p16 promoter region is widespread in CRC, and p16 methylation can be considered as an indicator of the prognosis of CRC [29].

Genome-wide hypomethylation is aberrant and involves early epigenetic changes during the development of CRC [30]. There are few studies into molecular events related to human genome hypomethylation, but there is evidence that gene-related specific hypomethylation changes are closely related to the development of CRC. In tumor cells, oncogenes are in a state of hypomethylation, and so they are activated. Abnormal hypomethylation of some genes, such as mothers against decapentaplegic homolog 3 (SMAD3), long interspersed nucleotide element-1 (LINE-1) and GDNF family receptor alpha 1 (GFRA1) genes, have been shown to be associated with the poor prognosis of CRC [31,32,33].

#### 2.1.2. DNA Hydroxymethylation in CRC

During active and passive demethylation pathways, there are several kinds of intermediates, such as 5-hydroxymethylcytosine (5hmC), 5-formylcytosine (5fC), 5-carboxylcytosine (5caC) and 5-hydroxymethyluracil (5hmU) [9]. Recently, DNA 5hmC is also found to be a biomarker for tumors and may play an important role during tumorigenesis [34]. 5hmC regulates intestinal differentiation in adults, and its aberrant alterations lead to the disorder of gene expression in CRC [35]. Besides, DNA 5hmC is associated with malignant tumor behavior in patients and is considered to be an independent prognostic factor for overall survival, indicating that down-regulation of DNA 5hmC may be a useful biomarker for prognostic assessment of CRC [36]. Aberrant hydroxymethylation of arachidonate 15-lipoxygenase (ALOX15), growth hormone releasing hormone receptor (GHRHR), tissue factor pathway inhibitor 2 (TFPI2) and transketolase like 1 (TKTL1) at some genome position is correlated with CRC [37]. These lines of evidence indicate that 5hmC in genomes may be a promising biomarker for the diagnosis and prognosis of CRC.

Abnormal 5hmC modifications often occur at transcription start site (TSSs) regions and proximal regions closer to TSSs than that of the abnormal methylation [37]. Some studies have shown that promoters with 5hmC have a natural resistance to hypermethylation in CRC [38]. This result means that 5hmC also functions as an important regulator during transcription, and can be used as a therapeutic target. Vitamin C can promote decitabine or azacytidine-induced DNA 5hmC, leading to subsequent reactivation of p21^WAF1^ (CDKN1A), an epigenetically silenced tumor suppressor in CRC [39]. Treatment with anti-cancerous omega-3 polyunsaturated fatty acids in CRC shows an increase in the genomic DNA 5hmC [40].

### 2.2. Histone Post-Translational Modification

Histone modification is mainly the various modifications of the free N-terminal, including acetylation, methylation, phosphorylation, ubiquitination and so on. These modifications direct the fate and function of histones, which further alter the chromosomal substructures and subsequently gene transcription. Histone modification can catalyze the change of gene expression through histone modification enzymes, including writers, erasers and readers, which play important roles in the occurrence and development of malignant tumors. Therefore, the dysregulation of histone modification in CRC may change the expression of corresponding genes. Histone modifications can result in the activation or inhibition of transcription, depending on the location and type of modification [41]. The acetylation of lysine residues is related to transcriptional activation and is considered an “activation marker” [42]. Studies have shown that the acetylation level of H3K27 in CRC is significantly higher than that in normal tissues [43]. Ashktorab et al. demonstrated that H3K12 and H3K18 acetylation are significantly increased in moderately and well-differentiated colon cancer, and are decreased in poorly differentiated colon cancer [44]. However, lysine methylation can cause many consequences, such as promotion of transcriptional activity, regulation of transcriptional inhibition and so on. H3K4me3 is enriched in transcriptionally active gene promoters, while H3K9me3 and H3K27me3 are present in transcriptional repressive gene promoters. Benard et al. demonstrated that the high expression of activated histone modification H3K4me3 and the low expression of silent modification H3K9me3 may be related to the poor clinical outcome of CRC [45].

Histone modification is dynamically regulated by a variety of enzymes. For example, histone methyltransferases (HMTs) and histone demethylases (HDMs) are responsible for increasing and removing methyl groups, respectively. Histone acetyltransferases (HATs) can increase the acetyl groups of lysine residues, while histone deacetylases (HDACs) can remove acetyl groups. The dynamic balance of these two enzyme activities maintains the proper state of histone acetylation. Therefore, the dysregulation of histone modification-related enzyme activities is related to the development of cancer [46]. For instance, H3K9 methyltransferase G9a promotes gastric cancer progression and suppresses its autophagy by activating mTOR signaling [47]. H4K16 acetyltransferase MYST1/KAT8 contributes to glioblastoma progression by activating EGFR signaling [48]. H3K9 deacetylase SIRT6, one of the class III HDACs, are important for melanoma and gliomblatoma progression via inhibition of the transcription of glycolytic genes [49,50]. In CRC, epigenetics also functions as a fundamental role during carcinogenesis and subsequent tumor development and metastasis. For instance, H3K79 methyltransferase DOT1L supports CRC cancer progression via epigenetically promoting c-Myc expression [51]. Histone demethylase JMJD2D can interacts with β-catenin, activating the transcription of its target genes, which further supports cell proliferation, migration, and invasion of CRC [52]. Histone deacetylase SIRT1 inhibits CRC metastasis by transcriptional repression of miR-15b-5p [53]. These results indicate that post-translational modifications of the histone are important for CRC tumorigenesis and may be used as therapeutic targets for targeted therapy. Importantly, inhibitors targeting histone deacetylase have become promising drugs for the treatment of CRC [54].

### 2.3. Non-Coding RNAs in CRC

#### 2.3.1. MicroRNAs in CRC

MiRNA is a type of non-coding single-stranded RNA molecule with a length of about 22 nucleotides encoded by endogenous gene. By binding to a specific site in the 3′ UTR region of the target gene’s mRNA, miRNA promotes the degradation of the target gene’s mRNA, thereby exercising the function of regulating gene expression. A large number of studies have shown that the abnormal regulation of miRNAs plays an important role in the development and metastasis of CRC, showing dual effects of promoting tumors or suppressing tumors [55]. Deng et al. proved that overexpression of miR-21 can promote cell proliferation and invasion, and can alleviate the inhibitory effect of the chemotherapy drug 5-fluorouracil (5-Fu), the first-line drug for the treatment of CRC, on the proliferation and invasion of HT-29 cells [56]. The low expression of miR-133b is related to the poor survival rate and metastasis of CRC [57]. In SW620 and HT-29 cell lines, the ectopic expression of miR-133b can effectively inhibit tumor cell proliferation and apoptosis in vitro and in vivo by directly targeting receptor tyrosine kinases [58]. It has been found that the expression of miR-143 and miR-145 is reduced in CRC. Transfection of miR-143 and miR-145 precursors into HCT116 or SW480 cell lines, respectively, can observe cell growth inhibition and drug sensitivity enhancement [59]. More and more evidence shows that miRNAs can be used as markers for prognostic evaluation and efficacy evaluation of CRC [6]. Therefore, inhibiting oncogenic miRNAs and restoring the tumor-suppressive miRNAs can be used as promising strategies for the treatment of CRC.

#### 2.3.2. LncRNAs in CRC

Long non-coding RNAs (lncRNAs) are transcripts longer than 200 nucleotides that do not encode proteins. They can interact with almost all molecules in cells, including RNA, DNA, proteins, and even metabolites. Recently, lncRNAs such as cancer-susceptibility 15 (CACS15), cytoskeleton regulator RNA (CYTOR), HOX transcript antisense RNA (HOTAIR), metastasis associated lung adenocarcinoma transcript 1 (MALAT1), taurine upregulated 1 (TUG1), nuclear paraspeckle assembly transcript 1 (NEAT1), miR-17-92a-1 cluster host gene (MIR17HG) and so on, have been shown to be tightly correlated with the prognosis of CRC and function as competitive endogenous RNAs (ceRNAs) [60]. These ceRNA networks, formed by the lncRNA/miRNA/mRNA interactions, have been found in a wide range of biological processes in CRC, including liver metastasis, epithelial-to-mesenchymal transition (EMT), inflammation, and chemotherapy/radiation resistance [60]. Moreover, lncRNAs also function as protein partners to regulate protein stabilization. For instance, lncRNA nuclear paraspeckle assembly transcript 1 (NEAT1) directly interacts with Ddx5 protein, enhancing its stability, and sequentially activating Wnt signaling in CRC [61]. LncRNA colon cancer associated transcript 1-L (CCAT1-L), which is specifically transcribed from the upstream 515kb site of MYC in human colorectal carcinoma, promotes long-term chromatin looping, which further enhances MYC transcription regulation via long-range interactions between the MYC promoter and its enhancers [62]. These findings indicate that lncRNAs may be potential biomarkers and therapeutic targets for CRC.

## 3. Cellular Metabolism in CRC

### 3.1. Aerobic Glycolysis in CRC

Current studies have found that the Warburg effect is closely related to the occurrence, invasion and metastasis of CRC [63,64]. Compared with normal mucosa, many metabolic pathways of CRC have changed. The metabolic changes of CRC is the result of metabolic reprogramming guided by the activation of proto-oncogenes and the inactivation of tumor suppressor genes [65]. Many genes and proteins related to glucose uptake and glycolysis are dysregulated in CRC, including K-RAS, hypoxia-inducible factor (HIF), MYC, PI3K/AKT/mTOR axis and their related signaling pathways and tumor suppressor gene p53 [66]. In fact, the dysregulation of many of the above genes is related to tumor aggressiveness and poor prognosis of CRC [67]. For instance, K-RAS mutations occur in approximately 40% of CRCs [68,69]. Yun et al. found that glucose transporter 1 (GLUT1) levels were up-regulated in the transcripts of CRC cell lines in the K-RAS gene mutation state, and K-RAS mutant cells showed increased glucose uptake and glycolysis [70]. Another major gene is HIF, which is stimulated by the hypoxic microenvironment. Up-regulation of HIF-1α expression was found in 55% of CRC biopsies [71]. HIF can up-regulate the expression of hundreds of genes, such as GLUT1, c-Myc, vascular endothelial growth factor (VEGF), and glycolysis-related genes. P53 is also one of the most import and mutant genes in CRCs. More than 40% of CRCs carry mutations in the tumor suppressor gene p53, leading to loss or gain of function [72]. Ma et al. found that the glycolysis of wild-type p53 CRC HCT116 cells contributes about 40% to ATP, while the contribution of p53 mutant cell glycolysis to ATP is up-regulated to about 66% [73].

### 3.2. Glutamine Metabolism in CRC

In addition to glycolysis, glutaminolysis is also important in CRC. Glutamine is the fastest consumed amino acid in tumor cells. The growth of cancer cells is dependent on glutamine, and tumor cells cannot grow in a medium lacking glutamine. After glutamine enters the cell via the transporter alanine-serine-cysteine transporter 2 (ASCT2), it is hydrolyzed into glutamate and ammonia under the action of glutaminase (GLS), glutamine can be converted into α-ketoglutaric acid (α-KG) and enter the tricarboxylic acid (TCA) cycle, which provides intermediate metabolites and energy for cells [74]. The gene expression of glutamine metabolism-related enzymes in CRC is up-regulated. For example, the level of GLS1 mRNA in CRC is significantly higher than that in neighboring normal tissues [75]. These changes are regulated by oncogenes and tumor suppressor genes. Among them, the proto-oncogene c-Myc is the main transcription factor that promotes the metabolism of glutamine in cancer cells. ASCT2 and GLS1 can be transcriptionally activated by c-Myc, thereby promoting the uptake and metabolism of glutamine [76,77].

### 3.3. Biosynthetic Metabolism in CRC

In addition to energy catabolism, the biosynthetic metabolism of CRC is also very different from normal human tissues, which is mainly reflected in lipids, proteins and nucleotides. For instance, comparing CRC to normal colon tissue, an integrated analysis of miRNA and mRNA endorses a twenty miRNAs signature, which is tightly correlated with metabolic genes including long-chain acyl-CoA synthetase 6 (ACSL6) and phosphoribosyl pyrophosphate synthetase 1 and 2 (PRPS1/2) [78]. Nucleotides in tumors are mainly synthesized by de novo pathways, and their de novo synthetase activities are relatively high in CRC [79,80].

The lipid metabolism of normal cells is maintained at a low level, and the activity of enzymes related to lipid metabolism is also low. Abnormal lipid metabolism of tumor cells is mainly manifested by de novo fatty acid synthesis and enhanced lipid synthesis [81]. Enzymes related to fatty acid synthesis have been discovered, such as ATP citrate lyase (ACLY), acetyl-CoA carboxylase (ACC) and fatty acid synthetase (FASN) is highly expressed in CRC [82,83]. Nevertheless, several genes, such as acetyl-CoA acyltransferase 2 (ACAA2), acyl-CoA dehydrogenase short chain (ACADS), acetyl-CoA acetyltransferase 1 (ACAT1), acyl-CoA oxidase 1 (ACOX), carnitine palmitoyltransferase 1A (CPT1A) and 3-hydroxy-3-methylglutaryl-CoA synthase 2 (HMGCS2), were shown to be downregulated in CRC, compared with distant normal colon tissue (NTC) in a transcriptome study [84]. This makes it complex for the relationship of lipogenesis and tumorigenesis in CRC.

Protein homeostasis is also important for cell fate. Interestingly, protein synthesis is also accurately modulated in cells and deregulation of it may be an important cause for carcinogensis. It is showed that the PI3K/AKT/mTOR signaling pathway is abnormally activated in CRC, which not only enhances glycolysis, but also enhances protein synthesis [85]. Activated mTOR can regulate downstream pathways, such as eukaryotic initiation factor 4E binding protein 1 (4EBP1) and S6 kinase 1 (S6K1), which are key regulators of protein translation [86].

## 4. Metabolism Influences Epigenetic in CRC

**Figure 1:**
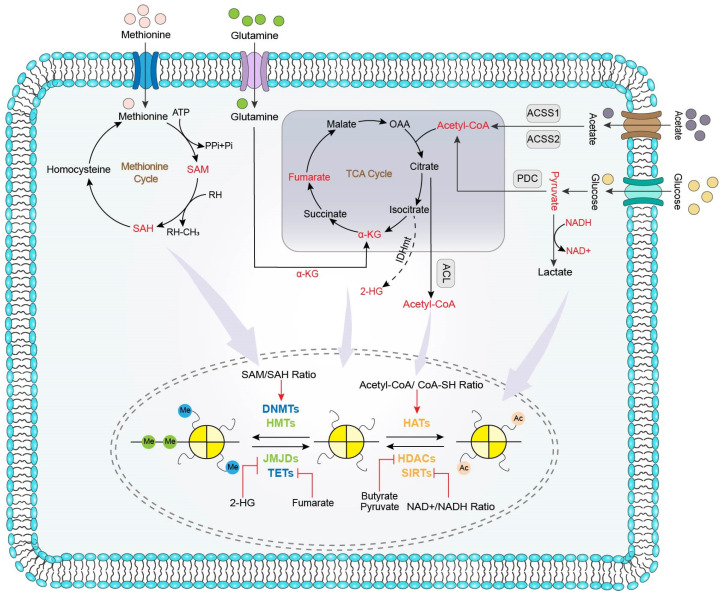
Mode of actions of metabolites modulating epigenetics in colorectal cancer.

As shown in Figure 1, metabolites can effluence epigenetics by modulating the epigenetic chemical reaction or regulating the enzymatic activity of epigenetic enzymes.

### 4.1. Metabolites Influences Epigenetic Process

#### 4.1.1. Methyl Donor Regulates Epigenetics in CRC

*S*-adenosylmethionine (SAM) is a product of the single-carbon metabolic cycle [87,88]. As the main biological methyl donor, SAM can methylate DNA, RNA, protein and phospholipids [89,90]. Therefore, it is considered to be a demethylation inhibitor that can induce gene hypermethylation and reverse overall hypomethylation. Wang et al. used exogenous administration of SAM to increase DNA methylation levels and block the growth cycle of SW620 and LoVo cell lines [91]. It is speculated that specific dietary interventions can affect the level of methylation in tumors [92]. Interestingly, this hypothesis has been confirmed in many studies. Methionine is an essential sulfur-containing amino acid, which reacts with ATP under the catalysis of adenosyltransferase to generate SAM. Methionine works synergistically with 5-Fu to inhibit the growth of CRC tumors, thereby affecting the level of methylation, disrupting nucleotide metabolism and redox balance [93]. There is a negative correlation between folate levels and the risk of CRC [94,95]. Folate metabolism can affect gene expression by regulating the level of SAM. Folate deficiency can lead to a decrease in SAM levels, resulting in DNA hypomethylation in colon cells [96,97]. However, conflicting results regarding the effect of folate status on DNA methylation have been reported. Another study showed that in a mouse model of CRC, a diet lacking folate reduced tumor size without affecting overall or gene-specific DNA methylation [98]. These findings may indicate that dietary folate levels are not the only determinant of DNA methylation status, and other confounding factors may regulate the role of folate as a methyl donor.

#### 4.1.2. Acetyl Donor Regulates Epigenetics in CRC

Acetyl-coenzyme A (acetyl-CoA) is a significant metabolic intermediate of the three major nutrients of glucose, fatty acid, and amino acid [88,99]. As the sole acetyl donor in acetylation, acetyl-CoA plays a key role in the process of histone acetylation [100]. Under “fed” or “fasted” state, acetyl-CoA levels fluctuate in cytosol/nucleus/mitochondria resulting in changes in protein acetylation levels [101]. It is reported that metabolic reprogramming of acetyl-CoA exists in many types of tumors [102]. In addition to glucose and other conventional carbon sources, it has recently been demonstrated that acetyl-CoA can be produced from acetate in a variety of cancers, including CRC [103]. Gao et al. found that acetate can produce acetyl-CoA, which affects the acetylation levels of H3K9, H3K27 and H3K56 in HT-29 cells [104]. Besides Acetate, butyrate can also up-regulate the level of Ac-CoA, thereby enhancing the histone acetylation of promoters of DNA mismatch repair genes [105]. Recent report also showed that acetyl-CoA synthetase 2 (ACSS2) is downregulated in CRC and is associated with poor tumor prognosis [106,107]. Chen et al. presented that histone H3 acetylation levels are significantly downregulated in CRC tissues compared to normal colon tissues [108]. These phenomena are directly related to changes in Ac-CoA levels.

### 4.2. Metabolites Influences the Activity of Epigenetic Enzyme

#### 4.2.1. Metabolites Affecting DNMTs and HATs

In CRC, increased levels of SAM can change the state of DNA hypomethylation and increase DNA methyltransferase activity [109,110]. In the SAM transmethylation reaction, *S*-adenosylhomocysteine (SAH) as a by-product is produced, which is a vigorous inhibitor of SAM-dependent methyltransferase [111,112]. Therefore, SAM/SAH ratios decide methyltransferase activity in vivo [88]. A present study shows that exogenous giving SAM increases SAM/SAH ratio, thus activating DNMTs and HATs resulting in increased methylation [113,114]. Dietary folate intake will change SAM/SAH ratio and cause fluctuations in DNMT levels, thereby affecting DNA hypomethylation in CRC [115,116,117,118].

#### 4.2.2. Metabolites Affecting JMJDs and TETs

In the metabolism of TCA cycle, isocitrate dehydrogenase (IDH) can catalyze the conversion of isocitrate to α-KG [119]. IDH gene mutations are closely related to the occurrence and development of tumors, but the mutation rate of IDH in CRC is only 1% [120,121]. When IDH is mutated, its enzymatic activity changes, which can convert α-KG into 2-hydroxyglutarate (2-HG), resulting in excessively high levels of 2-HG in the body [122]. 2-HG and α-KG are highly similar molecules. 2-HG can occupy the same binding pocket as α-KG, and competitively inhibit α-KG-dependent dioxygenase, including histone demethylases dependent of Jumonji C-domain-containing (JMJDs) and ten-eleven translocations (TETs) [123,124,125]. Gerecke et al. proved that Vitamin C and IDH inhibitor ML309 act synergistically in some way to mediate the displacement of 2-HG on the catalytically active site of TETs and reactivate its full functions, accompanied by an increase in overall DNA hydroxymethylation and apoptosis in mutated IDH cells (HCT116 IDH^R132H/+^) [126]. Fumarate and succinate, other metabolites of TCA cycle, can act as α-KG antagonists to inhibit α-KG-dependent dioxygenase activity [127]. It has been confirmed that the accumulation of succinate can promote the effect of Wnt/β-catenin signaling pathway in KRAS-mutant CRC by blocking TETs [128].

#### 4.2.3. Metabolites Affecting HATs

Both acetyl-CoA absolute levels and acetyl-CoA/CoA-SH ratios can regulate HATs activity in cancer cells [129,130]. Studies have shown that histone acetylation in mammalian cells relies on ACLY, which turns glucose-derived citric acid into acetyl-CoA. The effect of acetyl-CoA on HATs is regulated by the expression of ACLY [88]. Wellen et al. found that ACLY is the main source of acetyl-CoA for global histone acetylation under normal growth conditions, but ACLY gene silencing observably reduces the amount of histone acetylation in the HCT116 cell line [131].

#### 4.2.4. Metabolites Affecting HDACs and SIRTs

According to three-dimensional structures, functions, and sequence homology, HDACs can be roughly divided into four categories: metal-dependent class I, II and IV (classical HDACs) and NAD^+^-dependent class III (SIRT1-7) [132]. It is reported that butyrate and pyruvate can inhibit classical HDACs activity in CRC [133,134]. More and more studies have shown the role of butyrate in preventing CRC [135,136]. Butyrate accumulates in cancerous cells undergoing the Warburg effect. Hence, the increase of butyrate may inhibit the growth of CRC cells and act as an inhibitor of HDACs to up-regulate pro-apoptotic genes [137].

Nicotinamide adenine dinucleotide (NAD^+^) is a significant cofactor for the activity of SIRTs, which can directly and indirectly affect many key cell functions, including metabolic pathways [138]. Nicotinamide phosphoribosyltransferase (NAMPT) is up-regulated in different types of cancers such as CRC, resulting in an increase in the NAD(H) pool size and the NAD^+^/NADH ratio [139]. Recently, Brandl et al. confirmed the existence of c-Myc/NAMPT/SIRT1 feedback loop in CRC [140]. Among them, c-Myc can cause an increase in NAD^+^ to mediate the activity of SIRT1 through transcriptional activation of NAMPT. Studies have shown that MYC can also increase the activity of lactate dehydrogenase A (LDHA), thereby promoting the reduction of pyruvate to lactate by LDHA. At the same time, this process is accompanied by the oxidation of NADH to form NAD^+^ [141]. The high expression of SIRT3, SIRT5, and SIRT7 is associated with poor CRC prognosis, while SIRT2, SIRT4, and SIRT6 are the opposite [142,143,144,145,146,147].

## 5. Epigenetics Influence Metabolism in CRC

**Figure 2:**
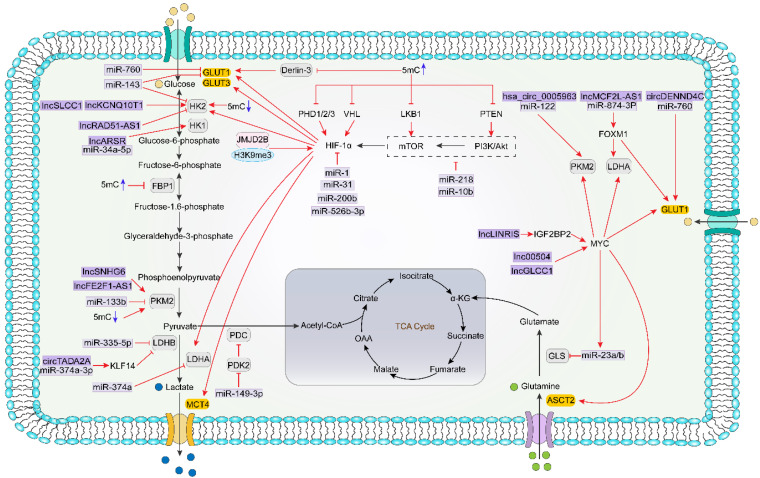
Mode of actions of epigenetics regulating metabolites in colorectal cancer.

As shown in Figure 2, epigenetics can modulate cellular metabolism in a direct or indirect manner. For direct modulation, epigenetic factors directly interact with genes of metabolism; for indirect modulation, epigenetic factors interact with major regulators of metabolism.

### 5.1. Epigenetics Direct Regulation of Metabolism-Related Genes

Epigenetic modification can directly change the mRNA expression of metabolic enzymes and transporters to regulate cell metabolism. DNA hypermethylation or hypomethylation in the promotor of glycolytic genes can directly determine their expression. Fructose-1,6-bisphosphatase-1 (FBP1) has been shown to be generally down-regulated in CRC [148]. It is considered that FBP1 is a key enzyme regulating gluconeogenesis, which can catalyze the hydrolysis of fructose-1,6-diphosphate to fructose-6-phosphate and inorganic phosphate. FBP1 can antagonize glycolysis, hence its low expression can increase glycolytic flux, which further promotes tumor proliferation and metastasis. Chen et al. found that in SW620, HCT116 and other CRC cell lines, DNA methylation mediates promoter methylation to silence FBP1 [149]. On the contrary, the up-regulation of hexokinase 2 (HK2) and pyruvate kinase isozyme 2 (PKM2) in CRC is beneficial to increase glycolytic flux by promoter hypomethylation, which further improves the Warburg effect and CRC tumorigenesis [150,151,152,153].

MiRNAs can also participate in the expression of metabolism-related genes at the post-transcriptional level to regulate the transformation of glycometabolism in CRC. MiRNAs regulate the expression of many genes involved in glutamate metabolism, including miR-23a/b targeting GLS [76], and glycolytic, such as miR-760 and miR-143 targeting GLUT1 [154,155], miR-1 and miR-143 targeting HK2 [156,157], miR-133b targeting PKM2 [158], miR-149-3p targeting PDK2 [159], miR-335-5p targeting LDHB [160] and miR-374a targeting LDHA [161].

LncRNAs also regulate cell metabolism in CRC via direct interaction with glycolytic genes, mRNAs and proteins through multiple molecular mechanisms. For instance, lncRNA SNHG6 could target the mRNA of PKM and induce hnRNPA1 to splice the mRNA of PKM specifically, resulting in an increase in the ratio of PKM2/PKM1 [162]. LncRNA FEZF1-AS1 could bind PKM2 protein and increase its stability, leading to an increase in both cytoplasmic and nuclear PKM2 levels. Increased cytoplasmic PKM2 promotes pyruvate kinase activity and aerobic glycolysis, whereas upregulated nuclear PKM2 further activates STAT3 signaling [163]. LncRNA KCNQ1OT1 promotes CRC progression by increasing aerobic glycolysis through stabilizing HK2 [164]. In addition, LncRNA RAD51-AS1 and SLCC1 can all target HK2 [165,166].

### 5.2. Epigenetics Indirectly Regulate Cellular Metabolism via Modulating Metabolism-Related Transcription Factors and Signaling Pathways

DNA methylation can silence major regulators including transcriptional factors such as HIF-1/2, c-Myc, p53, phosphatase and tensin homolog deleted on chromosome 10 (PTEN), PI3K/AKT and LKB1-AMPK that control cellular metabolism in CRC. It is reported that PTEN, LKB1, prolyl hydroxylase enzymes (PHD1/2/3, negative upstream factors of HIFs) are silenced by promoter hypermethylation in CRC [167,168,169]. Fu et al. proved that JMJD2B can be combined with HIF-1α to reduce H3K9me3 on the promoter of HIF-1α targeted genes, such as LDHA, GLUT1/3, HK2, von Hippel-Lindau (VHL) and monocarboxylate transporters 4 (MCT4), to up-regulate their expression and contribute to the malignant phenotype of CRC [170]. DNA methylation also silences the Derlin-3—a key protein that mediates proteasomal degradation of GLUT1 and a pivotal regulator of glucose transport—which is confirmed to be connected with the occurrence and prognosis of CRC [171,172].

Histone post-translational modifications in the promoter region of metabolic major regulator is also an important regulatory mode for epigenetics affecting cellular metabolism. For example, Fu et al. proved that JMJD2B can be combined with HIF-1α to reduce H3K9me3 on the promoter of HIF-1α-targeted genes to upregulate their expression and to contribute to the malignant phenotype of CRC [170].

Increasing researches have shown that miRNAs also exert an important influence on signal transduction through PI3K/AKT/mTOR, HIF-1α. For example, miR-1, miR-31, miR-526b-3p, miR-200b and miR-93-5p target HIF-1α and regulate its expression in CRC [156,173,174,175,176]. MiR-1 also targets Smad3 in CRC, leading to inhibition of glycolysis and cell proliferation [156]. Besides, the PI3K/AKT/mTOR signaling pathway is identified as a direct target of miR-218 and miR-10b [177,178].

LncRNA also indirectly regulates cellular metabolism in CRC. For instance, lncRNA GLCC1 promotes colorectal carcinogenesis and glucose metabolism by stabilizing c-Myc, protecting it from ubiquitination-mediated degradation [179]. As an important transcription regulator of c-Myc in CRC cells, lncRNA00504 regulates metabolism at the transcription level and affects a variety of metabolic pathways [180]. Wang et al. proved lncRNA LINRIS (long intergenic noncoding RNA for IGF2BP2 stability) stabilizes IGF2BP2, a newly found *N*6-methyladenosine (m^6^A) reader that can recognize c-Myc mRNA, via protecting it from autophagic degradation, thus promoting the expression of GLUT1, PKM2 and LDHA and enhancing aerobic glycolysis in CRC [181]. LncRNA MCF2L-AS1 (MCF2 transforming sequence-like protein antisense RNA 1) aggravates cell proliferation, invasion and glycolysis of CRC cells via sponging miR-874-3p that targets forkhead box protein M1 (FOXM1) [182].

Circular RNAs (circRNAs), a special kind of lncRNAs, often function as sponges to antagonize miRNAs. CircRNA circDENND4C also sponges miR-760, which further targets GLUT1 to facilitate cell proliferation, migration and glycolysis of CRC [154]. LncRNA ARSR sponges miR-34a-5p that targets HK1 to promote glycolysis, which further stimulates CRC invasion and metastasis [183]. Exosome-delivered circRNA hsa_circ_0005963 can sponge miR-122 targeting PKM2, thereby promoting glycolysis to induce chemoresistance in CRC [184]. CircRNA hsa_circ_0000231 and lncRNA HNF1A-AS1 can promote migration, invasion and aerobic glycolysis via inhibition of miR-124/MYO6 (myosin VI) axis in CRC [185,186]. However, the relationship between MYO6 and glycolysis is not clear. CircRNA TADA2A can inhibit aerobic glycolysis and suppress the progression of CRC via sponging miR-374a-3p, which targets Kruppel like factor 14 (KLF14), a transcriptional factor that can repress the expression of glycolytic enzyme LDHB [187].

## 6. Epigenetic-Metabolic Crosstalk as a Therapeutic Target of CRC

As shown in Table 1 and Table 2, many inhibitors of cellular metabolism and epigenetics are under investigation in CRC. These inhibitors are potential therapeutic drugs for the treatment of CRC in the future.

### 6.1. Metabolism Inhibitors

#### 6.1.1. Glycolysis Inhibitors

2-Deoxyglucose (2-DG) is a well-known glycolysis inhibitor. It has a similar structure to glucose and can competitively bind to HK2 with glucose, thereby stopping glycolysis. In HCT116 cells treated with 2-DG, the activity of acetyl-CoA is inhibited and the activity of HDACs is increased, and it is found that the degree of acetylation at multiple sites of histones H3, H4, H2A and H2B is significantly reduced [188]. In addition, 3-Bromopyruvate (3-BrPA) can also inhibit the activity of HK2, thereby inhibiting the production of ATP and inducing the death of CRC cells [189]. Hence, glycolysis is a viable target for regulating histone acetylation.

#### 6.1.2. Glutaminase Inhibitors

Bis-2-(5-phenylacetamido-1,2,4-thiadiazol-2-yl) ethyl sulfide (BPTES), CB-839, and compound 968 are glutaminase inhibitors that have been developed, and their inhibitory effects on CRC cell viability are compared. Compound 968 may be the most effective drug, because CRC cells are more sensitive to compound 968 than BPTES and CB-839 [190]. Compound 968 is a specific inhibitor of GLS1, which can inhibit the recombinant expression of GLS1, and combine with inactive GLS to prevent GLS1 activation. However, the safety and clinical efficacy of compound 968 for the targeted treatment of glutamine metabolism in CRC needs to be evaluated in further clinical trials. Therefore, targeting glutamine metabolism may be a promising method for the treatment of CRC.

#### 6.1.3. SAM Cycle Inhibitors

As an effective inhibitor of SAH hydrolase, 3-deazaneplanocin A (DZNep) inhibits DNA and histone methylation by reducing the SAM/SAH ratio in CRC [191].

### 6.2. Epigenetic Inhibitors

#### 6.2.1. DNMT Inhibitors

5-Azacytidine and 5-Aza-2′-deoxycytidine have been approved for clinical DNMT inhibitors by regulatory agencies in the United States and Europe. 5-Azacytidine and 5-Aza-2′-deoxycytidine have been clinically studied for metastatic CRC, and 5-Azacytidine is combined with a variety of drugs to treat CRC. Li et al. proved that 5-Aza-2′-deoxycytidine has a significant inhibitory effect on the growth of CRC cell lines HCT116 and SW620, and observed that DNMTs activity was significantly reduced after treatment with different concentrations of 5-Aza-2′-deoxycytidine [192]. However, these two DNMT inhibitors have great toxic and side effects, which may be related to the formation of a covalent bond between DNA and DNMT protein being acted on [202]. Unlike the previous two, zebularine has fewer side effects. Cheng et al., through the study of tumor cell lines such as HCT15, SW48, HT-29 and a variety of normal human fibroblast cell lines, found that zebularine shows high selectivity in inhibiting DNMTs [193]. In normal fibroblast cell lines, it has little effect on DNMT1, DNMT3a and DNMT3b enzymes, while in tumor cells, DNMT1 is almost completely inhibited, and the other two enzymes are also partially inactivated.

#### 6.2.2. HDAC Inhibitors

HDAC inhibitors can restore the activity and expression of silenced tumor suppressor genes by targeting HDAC, thereby inhibiting the proliferation of tumor cells and inducing their apoptosis. At present, drugs that have been clinically reviewed and approved, such as chidamide, belinostat, and valproic acid, have been studied and reported in the treatment of CRC. The above three drugs also have related reports on the basic research of CRC. Liu et al. found that chidamide can increase histone H3 acetylation and induce apoptosis of CRC cells, and these effects may be achieved by inhibiting PI3K/AKT and MAPK/RAS gene pathways [194]. Beck et al. proved that belinostat can induce hyperacetylation of histones and has an inhibitory effect on the proliferation of HCT116 cells [195]. Zhu et al. found that valproic acid can inhibit HDACs, induce HDAC2 degradation, and significantly reduce the number and average size of adenomas in the entire intestine of APC^min^ mice [196].

#### 6.2.3. SIRT Activators and Inhibitors

Since members of SIRTs play an important role in the regulation of cancer cell metabolism, specific inhibitors may provide a strategy for cancer cell metabolism. At present, there are many studies on SIRT inhibitors, and most of the inhibitors are aimed at inhibiting the deacetylase activity of SIRT1 and SIRT2 [203,204]. Ueno et al. found that tenovin-6, a small molecule inhibitor of SIRT1 and SIRT2, can induce apoptosis of CRC cells and enhance the anti-tumor properties of 5-Fu and oxaliplatin [197]. Interestingly, in addition to drugs, some nutrients also can be potential epigenetic modulators. For instance, SIRT6 is a tumor suppressor against glycolysis and has been shown to be 35 times activated by free fatty acids, such as myristic acid, oleic acid and linoleic acid [198].

#### 6.2.4. MiRNA Modulators

Suppressing miRNAs as oncogenes, and restoring the tumor suppressor function of miRNAs, are currently the actual strategies for cancer treatment. It is well known that the down-regulation of miRNAs that inhibit tumors contributes to the development of cancer. MiRNA-like molecules, such as miRNA mimics and miRNAs in encoding expression vectors, can effectively restore the functions of these “lost” miRNAs. MiRNA mimics are chemically modified double-stranded RNAs that can mimic endogenous precursor miRNAs and form active miRNA molecules after being processed in cells, thereby mediating the degradation and/or post-translational blocking of the target mRNA [205]. Using miRNA mimics to restore the down-regulated expression of miR-31, miR-335-5p and miRNA-802 can inhibit the proliferation, migration and invasion of CRC cells [160,199,200].

Besides, there are a variety of other methods, including antisense nucleotides and miRNA sponges, to inhibit miRNAs that are overexpressed in tumors. Antisense nucleotides are single-stranded oligonucleotides complementary to target miRNAs, which can block the interaction of miRNAs with their endogenous mRNA targets [206]. Valeri et al. proved that the specific inhibition of miR-135b can inhibit the proliferation, migration and induce apoptosis of CRC cells [201]. However, cancer treatment with miRNA as a target still faces many challenges. The temporary expression of miRNA mimics in cells also reduces the efficiency of treatment, and can cause innate immune responses, leading to adverse reactions. At the same time, the miRNA delivery system needs to be further improved [207].

In clinical practice, epigenetic drugs can be used not only alone, but also in combination with emerging anti-tumor drugs, which can greatly reduce the side effects of anti-tumor drugs while improving the efficacy. The combination of belinostat and irinotecan can synergistically enhance cell killing and inhibit tumor growth in CRC xenograft models [208]. Valproic acid can induce cell apoptosis and effectively enhance the cytotoxicity of bosutinib in vitro and in vivo at clinically relevant concentrations [209]. In addition, the current clinical studies of chidamide and valproic acid in the treatment of metastatic CRC are mainly based on combination drugs. Epigenetic drugs alone or in combination provide an attractive target for the treatment of CRC, but due to the lack of specificity and some adverse reactions, further research is still needed.

## 7. Summary

Abnormal epigenetic modifications in CRC can coordinate the dysregulation of driver gene expression. KRAS, HIF, MYC, PI3K/AKT/mTOR axis as driver genes are up-regulated in CRC that can increase glycolytic flux and lipid anabolism, which all contribute to the occurrence and development of CRC [66,210]. The crosstalk between epigenetic changes and metabolic reprogramming plays a very vital function in the occurrence and development of CRC, and it can be used as a promising target for tumor therapy.

In this review, we describe that DNA methylation, histone modification, and non-coding RNA-mediated epigenetic modification directly regulates the expression of metabolism-related genes in CRC, and can indirectly affect metabolic reprogramming through the regulation of metabolic-related signaling pathways. Metabolites such as glycolysis and TCA cycles can be used as cofactors, modification donors or antagonist molecules for epigenetic modifying enzymes in CRC to affect an epigenetic modification landscape. For example, SAM can be used as a methyl donor and can also activate DNMTs activity. Drugs targeting epigenetics and metabolism have achieved good results in CRC research. A deep understanding of the relationship between epigenetic modification and metabolic reprogramming is helpful in the discovery of new molecular targets and has great practical significance for the development of targeted drugs in CRC treatment.

At present, the research results of many drugs in CRC are based on cell culture systems, and to some extent it is difficult to represent the growth environment of cells in the body. The tumor microenvironment is of great significance to cell metabolism [211]. Devarasetty et al. created a colorectal tumor organism model to evaluate tumor microenvironmental effects by using its internal bioengineered mucosal cell body and embedded CRC HCT116 cells [212]. The use of the organism model provides a powerful tool for in vitro pharmacological testing, and also provides a good platform for the study of epigenetic and metabolic drugs in CRC. Future research should focus on exploring the complex crossover network between cell metabolism, epigenetic modification and tumor cell microenvironment, which is of great significance for the discovery of new or more effective molecular targets.

## Figures and Tables

**Table 1:** Drugs targeting cellular metabolism in CRC.

Inhibitor	Target Enzyme	Mode of Action	Ongoing Clinical Use/Trials	Ref.
2-Deoxyglucose (2-DG)	Hexokinases	It has a structure similar to glucose and can competitively bind to HK2 with glucose; it can also inhibit acetyl-CoA and increase the activity of HDACs	Phase I/II for prostate cancer, Phase I dose escalation trial	[188]
3-Bromopyruvate (3-BrPA)	Hexokinases	It can inhibit the activity of HK2, thereby inhibiting the production of ATP and inducing the death of CRC cells	NA	[189]
Compound 968	Glutaminases	It can inhibit the recombinant expression of GLS1 and combine with inactivated GLS to prevent GLS1 activation	NA	[190]
3-deazaneplanocin A (DZNep)	SAH hydrolase	It suppresses DNA and histone methylation by reducing the SAM/SAH ratio in CRC	NA	[191]

**Table 2:** Drugs targeting epigenetics in CRC.

Inhibitor	Target Enzyme	Mode of Action	Ongoing Clinical Use/Trials	Ref.
*DNMT inhibitors*				
5-Azacytidine, 5-Aza-2′-deoxycytidine, Zebularine	DNA methyltransferases	The first two drugs inactivate DNMTs non-selectively. Unlike the first two, zebularine has much smaller side effects and shows high selectivity in inhibiting DNMTs	5-Azacytidine (Phase I–III for various malignant tumors; Phase II for metastatic CRC), 5-Aza-2′-deoxycytidine (Phase I–III for various malignant tumors; Phase I for liver metastatic CRC;)	[192,193]
*HDAC inhibitors*				
Chidamide, Belinostat, Valproic acid	Histone deacetylases	HDAC inhibitors can induce histone acetylation and reverse the abnormal gene expression caused by HDACs	Chidamide, Valproic acid (Phase I–III for various malignant tumors; Phase II for metastatic CRC), Belinostat (Phase I/II for various malignant tumors; Phase I for CRC)	[194,195,196]
*SIRT activators and inhibitors*				
Tenovin-6	SIRT1, SIRT2	It inhibits the protein deacetylation activity of SIRT1 and SIRT2	NA	[197]
Myristic acid, Oleic acid, Linoleic acid	SIRT6	Free fatty acids activate SIRT6, which functions as a tumor suppressor to inhibit glycolysis	NA	[198]
*miRNA modulators*				
miRNA mimics, miRNAs in encoding expression vectors	miRNAs	They can effectively restore the functions of these “lost” miRNAs. For example, restoring the down-regulated expression of miR-31 can inhibit the proliferation, migration and invasion of CRC cells.	miR-16 mimic (Phase I for non-small cell lung cancer)	[160,199,200]
Antisense nucleotides, miRNA sponges	miRNAs	They can inhibit miRNAs that are overexpressed in tumors. For example, specific inhibition of miR-135b can inhibit the proliferation, migration and induce apoptosis of CRC cells.	NA	[201]

## Data Availability

This review did not contain any data.
